# Omecamtiv mercabil and blebbistatin modulate cardiac contractility by perturbing the regulatory state of the myosin filament

**DOI:** 10.1113/JP275050

**Published:** 2017-11-21

**Authors:** Thomas Kampourakis, Xuemeng Zhang, Yin‐Biao Sun, Malcolm Irving

**Affiliations:** ^1^ Randall Centre for Cell and Molecular Biophysics and British Heart Foundation Centre of Research Excellence King's College London London SE1 1UL UK

**Keywords:** cardiac myosin, muscle contraction, cardiac muscle regulation, fluorescence polarization, Omecamtiv Mecarbil

## Abstract

**Key points:**

- Omecamtiv mecarbil and blebbistatin perturb the regulatory state of the thick filament in heart muscle.
- Omecamtiv mecarbil increases contractility at low levels of activation by stabilizing the ON state of the thick filament.
- Omecamtiv mecarbil decreases contractility at high levels of activation by disrupting the acto‐myosin ATPase cycle.
- Blebbistatin reduces contractility by stabilizing the thick filament OFF state and inhibiting acto‐myosin ATPase.
- Thick filament regulation is a promising target for novel therapeutics in heart disease.

**Abstract:**

Contraction of heart muscle is triggered by a transient rise in intracellular free calcium concentration linked to a change in the structure of the actin‐containing thin filaments that allows the head or motor domains of myosin from the thick filaments to bind to them and induce filament sliding. It is becoming increasingly clear that cardiac contractility is also regulated through structural changes in the thick filaments, although the molecular mechanisms underlying thick filament regulation are still relatively poorly understood. Here we investigated those mechanisms using small molecules – omecamtiv mecarbil (OM) and blebbistatin (BS) – that bind specifically to myosin and respectively activate or inhibit contractility in demembranated cardiac muscle cells. We measured isometric force and ATP utilization at different calcium and small‐molecule concentrations in parallel with *in situ* structural changes determined using fluorescent probes on the myosin regulatory light chain in the thick filaments and on troponin C in the thin filaments. The results show that BS inhibits contractility and actin‐myosin ATPase by stabilizing the OFF state of the thick filament in which myosin head domains are more parallel to the filament axis. In contrast, OM stabilizes the ON state of the thick filament, but inhibits contractility at high intracellular calcium concentration by disrupting the actin‐myosin ATPase pathway. The effects of BS and OM on the calcium sensitivity of isometric force and filament structural changes suggest that the co‐operativity of calcium activation in physiological conditions is due to positive coupling between the regulatory states of the thin and thick filaments.

## Key points

- Omecamtiv mecarbil and blebbistatin perturb the regulatory state of the thick filament in heart muscle.
- Omecamtiv mecarbil increases contractility at low levels of activation by stabilizing the ON state of the thick filament.
- Omecamtiv mecarbil decreases contractility at high levels of activation by disrupting the acto‐myosin ATPase cycle.
- Blebbistatin reduces contractility by stabilizing the thick filament OFF state and inhibiting acto‐myosin ATPase.
- Thick filament regulation is a promising target for novel therapeutics in heart disease.

## Introduction

Contraction of heart muscle is driven by transient interactions between the myosin‐containing thick filaments and actin‐containing thin filaments, coupled to the hydrolysis of ATP. Each heartbeat is triggered by a transient rise in intracellular calcium concentration followed by calcium binding to the troponin complex in the thin filaments, which in turn induces a structural change in the thin filaments that allows the head or motor domain of myosin to interact with actin (Gordon *et al*. 2000). Recently, however, the canonical calcium–thin filament paradigm of contractile regulation has been extended in both cardiac and skeletal muscle by the emerging concept of regulatory states of the thick filament. That concept originates from structural studies of myosin and thick filaments from myosin‐regulated muscles, including vertebrate smooth muscle (Wendt *et al*. 2001) and invertebrate skeletal muscle (Woodhead *et al*. 2005; Alamo, 2008; Pinto *et al*. 2012), which are not regulated via their thin filaments, but by a change in the structure of the thick filaments controlled by phosphorylation of the myosin regulatory light chain (RLC) (Brito, 2011) or by calcium binding to its essential light chain (ELC) (Woodhead *et al*. 2013). In the OFF states of those thick filaments, the myosin head domains are prevented from either binding actin or hydrolysing ATP by a network of intra‐ and inter‐molecular interactions that stabilize a conformation in which they are folded back onto the myosin tails and the thick filament surface. Activation of these myosin‐regulated muscles releases the myosin heads from this OFF state, making them available for actin binding and ATP hydrolysis.

Multiple lines of evidence now indicate that this regulatory transition in the thick filament has been retained in muscle types and species that are regulated by the calcium–thin filament regulatory pathway to work in parallel with it (Linari *et al*. 2015; Fusi *et al*. 2016; Kampourakis *et al*. 2016). In resting skeletal and cardiac muscles of vertebrates, the myosin head domains lie in helical tracks similar to those seen in the OFF state of thick filaments from myosin‐regulated muscles (Huxley & Brown, 1967; Linari *et al*. 2015; Ait‐Mou *et al*. 2016). Many of the intra‐ and inter‐molecular interactions that stabilize that OFF structure are conserved in isolated thick filaments from mammalian heart muscle (Zoghbi *et al*. 2008; Al‐Khayat *et al*. 2013). Biochemical studies of vertebrate skeletal and cardiac muscle suggested that this OFF structure corresponds to a myosin state with very low ATP turnover called the ‘super‐relaxed’ state (Hooijman *et al*. 2011; Nogara *et al*. 2016b). Orientation‐sensitive probes on the myosin RLC in vertebrate skeletal and cardiac muscle cells show that this OFF state is destabilized by calcium activation, RLC phosphorylation, and by increased sarcomere length (Kampourakis *et al*. 2015, 2016; Fusi *et al*. 2016). Finally, a recent X‐ray diffraction study on intact trabeculae suggested a stress‐dependent activation of the thick filament structure (Reconditi *et al*. 2017).

**Figure 1:**
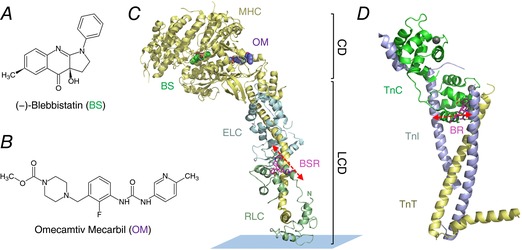
Labelling site of bifunctional sulforhodamine and binding sites of blebbistatin and omecamtiv mecarbil in the myosin head domain and labelling site of bifunctional rhodamine on troponin Chemical structures of (−)‐blebbistatin (BS) and omecamtiv mecarbil (OM) are shown in *A* and *B*, respectively. *C*, atomic model of squid myosin S1 in the detached conformation (PDB 1KK8). Bifunctional sulforhodamine (BSR) attached to the RLC (green) E‐helix is shown in pink, and BS and OM are shown in green and purple, respectively. The myosin heavy chain (MHC; CD‐catalytic domain; LCD‐light chain domain) and essential light chain (ELC) are shown in yellow and blue, respectively. The orientation of the BSR fluorescence dipole with respect to the motor domain is indicated by red dashed double arrow. The model was created by superimposition of the catalytic domains of myosin II bound to OM (PDB 4PA0) and BS (PDB 1YV3), with the catalytic domain of the squid myosin S1. *D*, labelling site of bifunctional rhodamine (BR, pink) in the troponin complex (PDB 1J1D). Troponin C, troponin I, troponin T and Ca^2+^ are shown in green, blue, yellow and grey, respectively. The BR probe is attached to the troponin C E‐helix. The orientation of the BR fluorescence dipole with respect to IT arm is indicated by red dashed double arrow. [Color figure can be viewed at wileyonlinelibrary.com]

Together, the results from these diverse approaches suggest that control of the structure of both the thin and thick filaments is required for the physiological regulation of cardiac contractility, including the strength and kinetics of the heartbeat. Thick filament regulation is likely to mediate the functional effects of phosphorylation of the myosin regulatory light chain (RLC) and myosin binding protein‐C, and probably length‐dependent activation and the Frank‐Starling relation (Colson *et al*. 2012; Ait‐Mou *et al*. 2016; Kampourakis *et al*. 2016). However, the molecular mechanisms underlying these effects remain poorly understood. Here we investigated those mechanisms using two small molecules that bind specifically to cardiac myosin: omecamtiv mecarbil (OM), a myosin activator in clinical trials for the treatment of heart disease (Malik *et al*. 2011; Teerlink *et al*. 2011; Liu *et al*. 2015), and (−)‐blebbistatin (BS), a widely used and well‐characterized myosin inhibitor (Kovacs *et al*. 2004; Dou *et al*. 2007; Farman *et al*. 2008). Both OM and BS bind to identified sites on the myosin head domain with micromolar affinity (Fig. 1) but have no known effects on other sarcomeric proteins. We determined the effects of OM and BS on the regulatory state of the thick filaments in cardiac muscle cells, in which the native conformation and organization of the myofilament sub‐proteome is preserved, using polarized fluorescence from bifunctional rhodamine probes on the myosin RLC (Kampourakis *et al*. 2014, 2015) (Fig. 1). To check the effects of OM and BS on the canonical calcium–thin filament regulatory pathway, we carried out a similar set of studies using a probe attached to troponin C. In both cases we made measurements over a range of calcium concentrations bracketing the physiological range, and correlated the changes induced by OM and BS in thick and thin filament structure with those on myocardial force in the same preparation and on myofibrillar ATPase activity.

The results show that the functional effects of OM and BS on cardiac contractility can be understood in terms of their effects on two regulatory pathways: the previously established effects on the acto‐myosin ATPase pathway and a novel effect on thick filament‐based regulation controlling the availability of myosin heads for contraction. OM stabilizes the ON state of the thick filament and myosin states with bound ADP (Malik *et al*. 2011; Liu *et al*. 2015; Rohde *et al*. 2017; Swenson *et al*. 2017), whereas BS stabilizes myosin states with bound ADP.P_i_ (Kovacs *et al*. 2004; Dou *et al*. 2007; Farman *et al*. 2008) and the OFF state of the thick filament. Moreover, our results give new insights into the physiological coupling between the regulatory states of the thick and thin filaments and establish mechanistic principles that may guide the development of new myosin modulators with improved therapeutic properties.

## Methods

### Animals and ethical approval

All animals were treated in accordance with the guidelines approved by the UK Animal Scientific procedures Act (1986) and European Union Directive 2010/63/EU. Wistar rats (male, 200–250 g) were killed by cervical dislocation (Schedule 1 procedure in accordance with UK Animal Scientific Procedure Act, 1986) and demembranated right ventricular trabeculae were prepared as described previously (Sun *et al*. 2009). All procedures were carried out in accordance with the guidelines of the Animal Welfare and Ethical Review Body (AWERB, King's College London). All animals have been kept with free access to food and water prior to use.

### Reagents

Omecamtiv mecarbil and (−)‐blebbistatin were purchased from Selleck (S2623) and Sigma (B0560), respectively. Stock solutions were prepared in DMSO (molecular biology grade, Sigma, D8418) and compound purities were estimated to be >95% by RP‐HPLC and ESI mass spectrometry.

### Preparation of BSR labelled cRLC and BR labelled cTnC

Bifunctional rhodamine labelled cTnC (BR‐cTnC‐E) and bifunctional sulforhodamine labelled cRLC (BSR‐cRLC‐E) were prepared as previously described (Sun *et al*. 2009; Kampourakis *et al*. 2014; Kampourakis & Irving, 2015).

### Reconstitution of labelled cRLCs and cTnCs into ventricular trabeculae

BR‐cTnC‐E was reconstituted into demembranated trabeculae by overnight soak in relaxing buffer (composition in mmol L^−1^: 25 imidazole, 15 disodium creatine phosphate (Na_2_CrP), 78.4 potassium propionate (KPr), 5.65 Na_2_ATP, 6.8 MgCl_2_, 10 K_2_EGTA, 1 DTT, pH 7.1) containing 0.5 mg ml^−1^ of BR‐cTnC‐E at 4°C, replacing about 80% of the endogenous cTnC (Sevrieva *et al*. 2014). The maximal calcium‐activated force after reconstitution with BR‐cTnC‐E was 40.7 ± 2.5 mN mm^−2^ (mean ± SEM, *n* = 11) at ∼2.0 μm sarcomere length.

BSR‐cRLC‐E was exchanged into demembranated trabeculae by extraction in CDTA‐rigor solution (composition in mmol L^−1^: 5 CDTA, 50 KCl, 40 Tris‐HCl pH 8.4, 0.1% (v/v) Triton X‐100) for 30 min followed by reconstitution with 40 μmol L^−1^ BSR‐cRLC‐E in relaxing solution for 1 h, replacing ∼50% of the endogenous cRLC (Kampourakis *et al*. 2014). The average maximal calcium‐activated force after cRLC exchange (37.4 ± 3.2 mN mm^−2^, mean ± SEM, *n* = 9) was 85 ± 7% of that before cRLC exchange.

### Fluorescence polarization experiments

Composition of experimental solutions and activation protocols were identical to those described previously for fluorescence polarization experiments (Sun *et al*. 2009; Kampourakis *et al*. 2014; Kampourakis & Irving, 2015). Polarized fluorescence intensities were measured as described previously for skeletal and cardiac muscle fibres (Corrie *et al*. 1999; Brack *et al*. 2004; Sun *et al*. 2009; Kampourakis *et al*. 2014). Fluorescence emission from BSR‐cRLC‐E and BR‐cTnC‐E in trabeculae was collected by a 0.25 NA objective using an excitation light beam in line with the emission path. The polarization of the excitation and emitted beams was set either parallel or perpendicular to the trabecular axis, allowing determination of the order parameter <*P*_2_> that describes the dipole orientations in the trabeculae (Dale *et al*. 1999).

The sarcomere length of trabeculae was measured by laser diffraction in relaxing solution prior to each activation. Activating solution contained (in mmol L^−1^): 25 imidazole, 15 Na_2_CrP, 58.7 KPr, 5.65 Na_2_ATP, 6.3 MgCl_2_, 10 CaCl_2_, 10 K_2_EGTA, 1 DTT, pH 7.1. Each activation was preceded by a 2‐min incubation in pre‐activating solution (composition in mmol L^−1^: 25 imidazole, 15 Na_2_CrP, 108.2 KPr, 5.65 Na_2_ATP, 6.3 MgCl_2_, 0.2 K_2_EGTA, 1 DTT, pH 7.1). Solutions with varying concentrations of free [Ca^2+^] were prepared by mixing relaxing and activating solutions using MAXCHELATOR software (maxchelator.stanford.edu). Isometric force and steady‐state fluorescence polarization values were measured once steady force had been established. The dependence on force and order parameters on free calcium concentration was fitted to data from individual trabeculae using non‐linear least‐squares regression to the modified Hill equation:

F=Y0+A×[Ca2+]nH/(− lo g10[ pC a50]nH+[Ca2+]nH),

where pCa_50_ is the negative logarithm of [Ca^2+^] corresponding to half‐maximal change in *F*, *n*_H_ is the Hill coefficient, *Y*_0_ is the baseline, and *A* is the amplitude (for normalized force data: *Y*_0_ = 0 and *A* = 1). Trabeculae which showed a decline in maximal calcium‐activated force of more than 15% after the pCa titrations were discarded.

Compound stocks were directly diluted in physiological buffers used for trabeculae experiments. The DMSO concentration was typically not more than 0.2% (v/v) and control experiments showed that addition of 0.2% (v/v) DMSO had no effect on calcium sensitivity or cooperativity of force and probe orientation. Prior to the experiments, demembranated rat ventricular trabeculae were incubated in relaxing solution containing the appropriate concentration of either omecamtiv mecarbil or blebbistatin for 25–30 min at 22°C.

### Preparation of cardiomyofibrils and ATPase activity measurements

Cardiomyofibrils (CMFs) were prepared by homogenizing freshly frozen ventricular tissue samples in myofibril buffer (composition in mmol L^−1^: 20 imidazole pH 7.4, 75 KCl, 2 MgCl_2_, 2 EDTA, 1 DTT, 1% (v/v) Triton X‐100, protease inhibitor cocktail (Roche), PhosStop cocktail (Roche)) followed by centrifugation at 5000 *g* for 5 min at 4°C. CMFs were washed and homogenized three more times in the same buffer without Triton X‐100.

CMFs were washed three times in ATPase assay buffer (composition in mmol L^−1^: 20 MOPS pH 7.0, 35 NaCl, 5 MgCl_2_, 1 EGTA, 1 DTT) with varying concentrations of CaCl_2_ (pCa 9 to pCa 4.3) and the CMF concentration adjusted to 0.5 mg ml^−1^ (Utter *et al*. 2015). For ATPase measurements at activating Ca^2+^ concentrations, CMFs were partially crosslinked with 5 mmol L^−1^ *N*‐hydroxysuccinimide (NHS) and 2 mmol L^−1^ 1‐ethyl‐3‐(3‐dimethylaminopropyl)carbodiimide hydrochloride (EDC) in myofibril buffer on ice for 90 min (Herrmann *et al*. 1993). The crosslinking reaction was stopped by the addition of 25 mmol L^−1^ glycine pH 8.0 and 10 mmol L^−1^ DTT for 30 min on ice and CMFs processed for experiments as mentioned above. Chemical crosslinking prevents CMFs from shortening during calcium activation. Reactions were started by the addition of 2.5 mmol L^−1^ ATP and samples taken at the indicated time points were quenched with 0.5 volumes ice cold 25% (w/v) TCA solution. Samples were kept on ice at all times, diluted with double‐deionized water and inorganic phosphate content measured using the malachite green assay according to manufacturer's instructions (Sigma, MAK030).

### Statistical analysis

All data sets were normally distributed as assessed by Shapiro‐Wilk test (*P* > 0.05). Statistical significance of difference between groups was assessed with a one‐way ANOVA followed by Turkey's *post hoc* test. Paired data sets were analysed by a two‐tailed paired Student's *t* test. Details of significance levels are shown in the figure captions.

## Results

### Effects of omecamtiv mecarbil and blebbistatin on isometric force production in rat ventricular trabeculae

**Figure 2:**
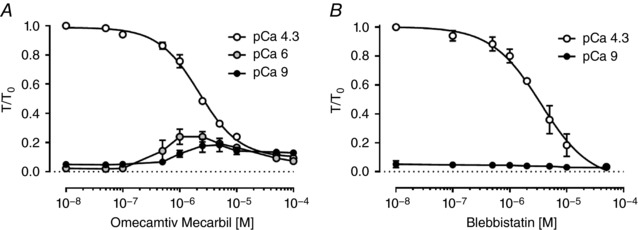
Concentration dependence of the effects of omecamtiv mecarbil (OM) and blebbistatin (BS) on isometric force development of native demembranated rat right ventricular trabeculae *A* and *B*, dose–response curves for OM and BS, respectively, on passive force at pCa 9 (filled circles), and active force at pCa 6 (grey circles) and pCa 4.3 (open circles). Isometric forces were normalized to that at pCa 4.3 in the absence of drugs (*T*_0_). Values indicate means ± SEM (*n* = 3). BS reduced passive force at pCa 9 (filled circles) by ∼60% with an EC_50_ of 2.3 ± 0.9 μmol L^−1^.

Although omecamtiv mecarbil (OM) is generally regarded as an activator of cardiac myosin, and OM increases cardiac output under therapeutic conditions (Malik *et al*. 2011), its effects depend strongly on calcium concentration [Ca^2+^] and are inhibitory at high [Ca^2+^] (Fig. 2 *A*). OM does activate isometric force in isolated trabeculae both at very low [Ca^2+^] (pCa 9, Fig. 2, black circles), and also around 1 μmol L^−1^ [Ca^2+^], close to the physiological value at systole under basal conditions. The largest effects were observed at micromolar concentrations of OM, and the increases in isometric force were statistically significant (*P* < 0.05) for [OM] = 1 μmol L^−1^ and higher. Isometric force was lower at higher OM concentrations, as reported previously for isolated rat cardiomyocytes (Nagy *et al*. 2015). In contrast, OM strongly inhibited force production at full calcium activation (pCa 4.3, Fig. 2 *A*, open circles) with an EC_50_ of 2.12 ± 0.17 μmol L^−1^ (mean ± SEM, *n* = 3), in agreement with its reported affinity for isolated β‐cardiac myosin S1 (Malik *et al*. 2011). Interestingly, the inhibition of maximal active isometric force has not been observed in human myocardium for [OM] ≤ 10 μmol L^−1^ (Swenson *et al*. 2017). OM slowed the rate of isometric force development at all [Ca^2+^] (data not shown), consistent with previous studies on isolated rodent cardiomyocytes (Nagy *et al*. 2015).

In contrast, blebbistatin (BS) inhibited isometric force in ventricular trabeculae at all [Ca^2+^] (Fig. 2 *B*). At full calcium activation (pCa 4.3) force inhibition by BS had an EC_50_ of 3.17 ± 0.43 μmol L^−1^, consistent with the binding affinity of BS for isolated skeletal myosin S1 in the ADP.P_i_ state (Kovacs *et al*. 2004) and with the EC_50_ for force inhibition by BS in mouse papillary muscle (∼2.8 μmol L^−1^) (Dou *et al*. 2007). BS also reduced passive force slightly at pCa 9, with a similar EC_50_ (Fig. 2 *B*). BS did not significantly affect the apparent rate of isometric force development (data not shown).

### Omecamtiv mecarbil and blebbistatin induce changes in thick filament structure

We determined the effect of OM and BS on thick filament structure in trabeculae using a bifunctional sulforhodamine (BSR) probe attached to the myosin regulatory light chain (cRLC, Fig. 1). The probe was cross‐linked to the cRLC E‐helix (BSR‐cRLC‐E), which is almost parallel to the long axis of the myosin light chain domain (LCD) (Kampourakis *et al*. 2015). The orientation of the RLC region of the myosin heads with respect to the thick filament axis can be determined from the polarization of the fluorescence from this probe. The results are expressed in terms of the order parameter *<P_2_>* (Dale *et al*. 1999), which would be +1 if all the probe dipoles (red arrow in Fig. 1) are parallel to the trabecular or thick filament axis, and −0.5 if they are perpendicular. *<P_2_>* decreases when trabeculae are activated, indicating a more perpendicular orientation of the cRLC E‐helix and LCD with respect to the thick filament axis, and a more ON state of the thick filament.

**Figure 3:**
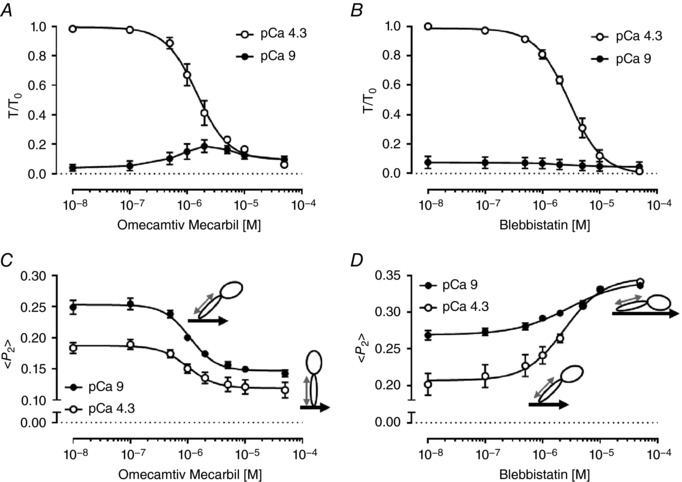
Concentration dependence of the effect of omecamtiv mecarbil and blebbistatin on force and myosin head orientation measured from BSR‐cRLC‐E exchanged trabeculae *A* and *C*, effect of OM on isometric force (*A*) and order parameter <*P_2_*> (*C*) for BSR‐cRLC‐E at pCa 9 (closed circles) and pCa 4.3 (open circles). *B* and *D*, concentration‐dependent effect of BS on isometric force (*B*) and order parameter <*P_2_*> (*D*) at pCa 9 (closed circles) and pCa 4.3 (open circles). Note that the effects of OM and BS on isometric force are identical to those for native, untreated trabeculae (Fig. 1). Values indicate means ± SEM (*n* = 4). The pictograms indicate the orientation of BSR probe (grey double arrow) and myosin heads with respect to the thick filament axis (black thick arrow). The angular changes are exaggerated for clarity.

Incubating relaxed trabeculae in OM produced a decrease in *<P_2_>* for the cRLC E‐helix probe (Fig. 3 *C*, pCa 9, filled circles), with an EC_50_ of 1.12 ± 0.09 μmol L^−1^ (mean ± SEM, *n* = 4), and a Hill coefficient of 1.75 ± 0.14 (mean ± SEM, *n* = 4), indicating positive cooperativity. The maximum effect of OM on *<P_2_>* was ∼70% larger than that of maximal calcium activation of ventricular trabeculae in the absence of drug. OM also reduced *<P_2_>* during maximal calcium activation (pCa 4.3, open circles), with a similar EC_50_ (1.03 ± 0.14 μmol L^−1^). These EC_50_ values are similar to those for active isometric force of native and BSR‐cRLC‐E exchanged trabeculae (Figs 2 *A* and 3 *A*), and to that of the ATPase rate of isolated β‐cardiac myosin (Malik *et al*. 2011). However, whilst addition of OM was associated with a more ON thick filament (a lower *<P_2_>* indicating that the LCD of the myosin heads is more perpendicular to the filament axis) in both relaxing and activating conditions (Fig. 3 *C*), it had opposite effects on isometric force in these two conditions (pCa 9 and pCa 4.3; Fig. 3 *A*). Moreover, there was no sign in the *<P_2_>–*[OM] relation (Fig. 3 *C*) of the bell‐shaped dose–response relation of force at low [Ca^2+^] (pCa 9, Figs 2 *A* and 3 *A*). Thus, although OM binds stoichiometrically to a single site on the myosin catalytic domain (CD) (Malik *et al*. 2011; Winkelmann *et al*. 2015), its effect on contractility, in contrast with that on cRLC E‐helix orientation, depends strongly on [Ca^2+^].

In contrast to OM, incubation of trabeculae in BS increased <*P_2_*> for the BSR‐cRLC‐E probe, indicating that BS stabilizes the OFF conformation of the thick filament in which the LCD of the myosin heads is more parallel to the filament axis (Kampourakis *et al*. 2014, 2015). BS increased <*P_2_*> at both low and high [Ca^2+^], with EC_50_ values of 2.91 ± 0.78 μmol L^−1^ and 2.33 ± 0.38 μmol L^−1^, respectively (Fig. 3 *D*), similar to those for passive and active force in the same trabeculae (Fig. 3 *B*) and in trabeculae containing the native cRLC (Fig. 2 *B*). The change in cRLC E‐helix orientation associated with calcium activation was attenuated by BS (Fig. 3 *D*), and became insignificant at [BS] = 5 μmol L^−1^, despite the fact that active isometric force (*T*/*T*_0_) at this BS concentration is still 31 ± 7% (mean ± SEM, *n* = 4) of its control value (Fig. 3 *B*).

### Effect of omecamtiv mecarbil and blebbistatin on cardiomyofibrillar ATPase activity

To better understand the mechanisms underlying the ability of OM to either activate or inhibit isometric force depending on the conditions, we measured the ATPase activity of freshly prepared rat ventricular cardiomyofibrils at 50 μmol L^−1^ OM, a concentration at which the thick filament structure is fully ON, but calcium‐activated force is largely inhibited (Fig. 3). For comparison, we also measured the effects on myofibrillar ATPase of 50 μmol L^−1^ BS, a concentration that induces the OFF state of the thick filament and also inhibits calcium‐activated force.

**Figure 4:**
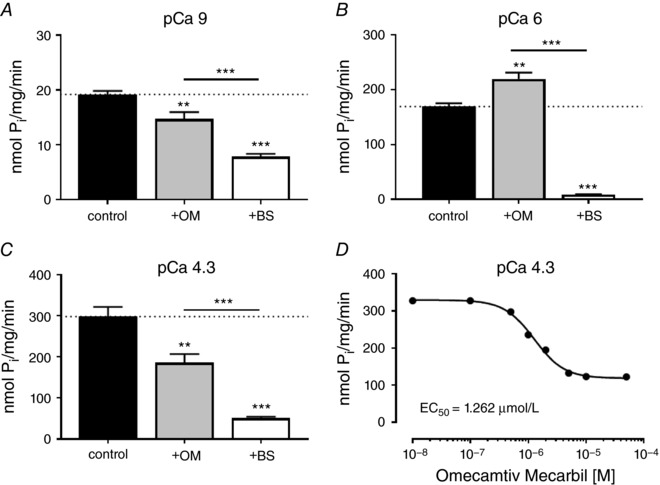
Effects of omecamtiv mecarbil and blebbistatin on the ATPase activity of cardiomyofibrils *A*–*C*, steady‐state ATPase rates for control, OM treated and BS treated CMFs at pCa 9 (*A*), pCa 6 (*B*) and pCa 4.3 (*C*). Values indicate means ± SEM (*n* = 4–8). *D*, dose–response curve for the effect of omecamtiv mecarbil on steady‐state cardiomyofibriliar ATPase activity at pCa 4.3. Statistical significance of differences between groups were assessed by a one‐way ANOVA with Turkey's *post hoc* test: ^**^ *P* < 0.01; ^***^ *P* < 0.001.

Addition of 50 μmol L^−1^ OM inhibited the cardiomyofibrillar ATPase rate at both low (pCa 9) and high [Ca^2+^] (pCa 4.3) (Fig. 4, grey bars). OM reduced the ATPase of relaxed myofibrils by about 24%, and the ATPase rate of isometrically contracting myofibrils at pCa 4.3 by about 37% with an EC_50_ of ∼1.3 μmol L^−1^ (Fig. 4 *D*), consistent with its effects on thin filament‐activated ATPase activity of isolated porcine heavy meromyosin (HMM) (Liu *et al*. 2015) and the actin‐activated ATPase activity of recombinant human β‐cardiac myosin S1 (EC_50_ ∼0.5 μmol L^−1^) (Swenson *et al*. 2017). Thus, although addition of 50 μmol L^−1^ OM at low and high [Ca^2+^] induces a more ON thick filament structure (Fig. 2 *C*) and activates isometric force at low [Ca^2+^] (Figs 2 *A* and 3 *A*), it *inhibits* the ATPase under the same conditions. Interestingly, the fractional inhibition of myofibrillar ATPase at maximal calcium activation (∼37%) is much less than that of isometric force (∼90%). However, OM increases the ATPase activity of cardiac myofibrils at intermediate levels of activation (pCa 6) (Fig. 4 *B*), similar to its effect on isometric force under the same conditions (Fig. 2 *A*). Moreover, the similar EC_50_ for OM's effect on myosin head orientation (Fig. 3 *C*) and ATPase activity suggests coupling between biochemical and structural states of myosin.

Addition of 50 μmol L^−1^ BS reduced the ATPase rate of cardiac myofibrils at all [Ca^2+^] tested by 60–90%, as expected from the inhibitory effects of BS on isometric force and thick filament structure (Fig. 3 *B* and *D*).

### Omecamtiv mecarbil and blebbistatin have contrasting effects on the calcium sensitivity of isometric force and thick filament structure

The activating effect of OM on isometric force at low and intermediate [Ca^2+^] implies that it sensitizes the myofilaments to calcium, an effect that might be associated with the more ON thick filament structure induced by the drug, although in apparent contradiction with its inhibition of myofibrillar ATPase at high [Ca^2+^]. To understand better the action of OM on the calcium sensitivity of contractile activation, we determined the effect of a clinically relevant concentration of OM of 1 μmol L^−1^, which is within the range of plasma concentrations measured during clinical trials (0.25–2.5 μmol L^−1^) (Cleland *et al*. 2011; Teerlink *et al*. 2011, 2016; Greenberg *et al*. 2015), on the [Ca^2+^] dependence of myocardial force and thick filament structure in the physiological range. As before we contrasted the effects of OM with those of BS, at a concentration (5 μmol L^−1^) that induced a largely OFF state of the thick filament, but left enough isometric force to allow us to determine its [Ca^2+^] dependence.

**Figure 5:**
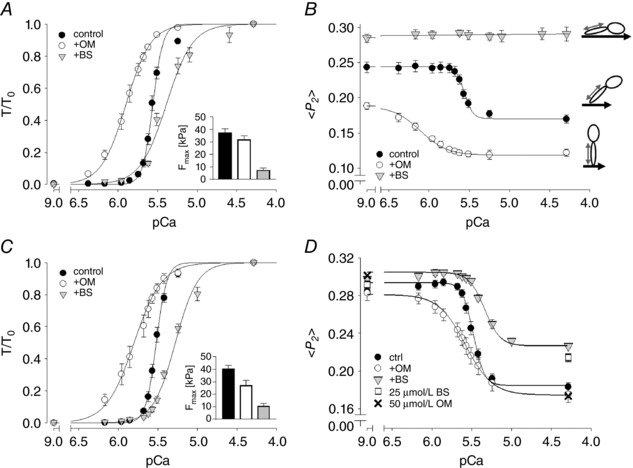
Calcium dependence of force, and cRLC E‐helix and cTnC E‐helix orientation in the absence (filled circles), and presence of 1 μmol L^−1^ omecamtiv mecarbil (+OM, open circles) and 5 μmol L^−1^ blebbistatin (+BS, grey triangles) *A* and *C*, normalized force–pCa relation for BSR‐cRLC‐E and BR‐cTnC‐E exchanged trabeculae, respectively. The maximal forces at pCa 4.3 are shown in the inset. *B* and *D*, <*P_2_*>–pCa relation measured in parallel with force for BSR‐cRLC‐E and BR‐cTnC‐E exchanged trabeculae, respectively. Means ± SEM (*n* = 4–9). Pictograms indicate the orientation of BSR probe (grey double arrow) and myosin heads with respect to the thick filament axis (black thick arrow)

**Figure 6:**
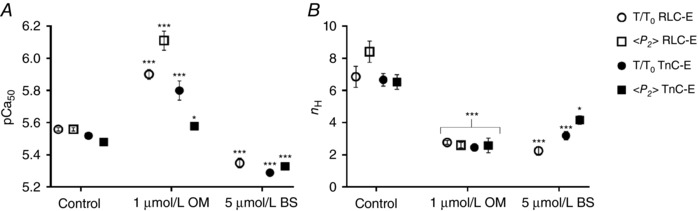
Summary of force–pCa (circles) and <*P_2_*>–pCa (squares) parameters *A* and *B*, pCa_50_ (*A*) and *n*_H_ values (*B*) for BSR‐cRLC‐E (open symbols) and BR‐cTnC‐E (closed symbols) exchanged trabeculae. Means ± SEM (*n* = 4–9). Statistical significance of differences from the control group was assessed by a one‐way ANOVA with Turkey's *post hoc* test: ^*^ *P* < 0.05; ^***^ *P* < 0.001.

At 1 μmol L^−1^ OM had a large effect on the [Ca^2+^] dependence of isometric force (Fig. 5 *A*, open circles), particularly in the physiologically relevant range around pCa 6. The pCa required for half‐maximal force (pCa_50_, Fig. 6 *A*, Table 1) increased from 5.56 ± 0.01 (mean ± SEM, *n* = 9) in the absence of OM to 5.90 ± 0.03 in its presence, but the Hill coefficient (*n*_H_, Fig. 6 *B*), a measure of the cooperativity of calcium activation, decreased from 6.85 ± 0.65 to 2.78 ± 0.12. The effects of 1 μmol L^−1^ OM on the calcium sensitivity of thick filament structure as reported by the cRLC E‐helix probe were even larger (Fig. 6 *B*). pCa_50_ for probe orientation increased from 5.56 ± 0.01 (the same as the value for force in the absence of OM) to 6.11 ± 0.06, and *n*_H_ decreased from 8.41 ± 0.66 to 2.61 ± 0.17. The [Ca^2+^] dependence of the orientation of the cRLC E‐helix probe in the absence of OM is similar to that reported previously for a BSR probe on the N‐lobe of the cRLC (Kampourakis *et al*. 2014).

**Table 1:** Summary of force–pCa and <*P_2_*>–pCa parameters for BSR‐cRLC‐E and BR‐cTnC‐E exchanged trabeulae

		BSR‐cRLC‐E			BR‐cTnC‐E		
		Control	+ 1 μmol L^−1^ OM	+ 5 μmol L^−1^ BS	Control	+ 1 μmol L^−1^ OM	+ 5 μmol L^−1^ BS
	*n*	9	5	4	11	5	4
Force	*F*_max_ (kPa)	37.4 ± 3.2	32.0 ± 3.0	7.4 ± 3.5$$$	40.7 ± 2.5	27.2 ± 4.3$	11.1 ± 2.6$$$
	pCa_50_	5.56 ± 0.01	5.90 ± 0.03$$$	5.35 ± 0.03$$$	5.52 ± 0.01	5.80 ± 0.06$$$	5.29 ± 0.02$$$
	*n*_H_	6.85 ± 0.65	2.78 ± 0.12$$$	2.25 ± 0.26$$$	6.66 ± 0.39	2.47 ± 0.22$$$	3.20 ± 0.26$$$
<*P_2_*>	pCa_50_	5.56 ± 0.01	6.11 ± 0.06$$$ ^,^ **	—	5.48 ± 0.01	5.58 ± 0.02$ ^,^ *	5.33 ± 0.02$$$ ^,^ *
	*n*_H_	8.41 ± 0.66	2.61± 0.17$$$	—	6.53 ± 0.45	2.59 ± 0.46$$$	4.17 ± 0.26$ ^,^ *
	<*P_2_*> ‐ amplitude (*A*)	0.074 ± 0.007	0.070 ± 0.002	—	0.109 ± 0.003	0.107 ± 0.007	0.081 ± 0.002$$
	<*P_2_*> ‐ baseline pCa 4.3 (*Y*_0_)	0.171 ± 0.006	0.119 ± 0.004$$$	—	0.185 ± 0.005	0.175 ± 0.008	0.226 ± 0.002$$$

Values indicate means ± SEM. Statistical significance of differences between groups was assessed by a one‐way ANOVA with Turkey's *post hoc* test: ^$^ *P* < 0.05; ^$$^ *P* < 0.01, ^$$$^ *P* < 0.001. Statistical significance of differences within groups was assessed with a paired two‐tailed Student's *t* test: ^*^ *P* < 0.05; ^**^ *P* < 0.01

In marked contrast with the effects of OM, 5 μmol L^−1^ BS *decreased* pCa_50_ for normalized force by ∼0.25 pCa units (Fig. 5 *A*, inverted triangles; Fig. 6 *A*). BS, like OM, reduced *n*_H_ to ∼2.5 (Fig. 6 *B*; Table 1). Thick filament structure as reported by the RLC probe was independent of [Ca^2+^] at 5 μmol L^−1^ BS (Figs 5 *B* and 3 *D*). Strikingly, the [Ca^2+^] dependences of force and thick filament structure in the presence of OM and BS (Fig. 5 *A* and *B*; open circles, inverted triangles) bracket the control relationship (filled circles). OM produces a more ON thick filament structure and sensitizes force to calcium; BS does the opposite. However both OM and BS greatly reduce the co‐operativity of calcium activation (Fig. 6 *B*), suggesting that the steep co‐operativity in control conditions requires a thick filament structure that is finely poised between the OFF and ON states.

### Effects of omecamtiv mecarbil and blebbistatin on *thin* filament activation

The correlation described above, between the changes in thick filament structure induced by OM and BS and the calcium dependence of active force mediated by Ca^2+^ binding to troponin in the thin filaments, suggests that the activation state of the thin filament is sensitive to that of the thick filament (Kampourakis *et al*. 2016). To further investigate that possibility we used a bifunctional rhodamine (BR) probe on the E‐helix of cardiac troponin C (cTnC) in the so‐called ‘IT arm’ of troponin to determine directly the effects of OM and BS on the activation state of the thin filament (Sun *et al*. 2009; Kampourakis *et al*. 2014).

The effects of OM (1 μmol L^−1^) and BS (5 μmol L^−1^) on isometric force in trabeculae containing BR‐cTnC‐E (Fig. 5 *C*) were similar to those reported above. However, in contrast to their effects on the activation state of the thick filament, neither OM nor BS changed the activation state of the thin filament as reported by the cTnC E‐helix probe in relaxing conditions (pCa 9) (Fig. 5 *D*). Since 1 μmol L^−1^ OM activates isometric force in these conditions (Figs 2 *A* and 3 *A*), this is a surprising result. To check it, we measured the orientation of the cTnC E‐helix probe at a series of OM concentrations up to 50 μmol L^−1^ and found no effect of OM on probe orientation in either relaxing conditions (pCa 9) or during maximal calcium activation (pCa 4.3) (crosses in Fig. 5 *D*), in contrast with its effect on thick filament structure in the same conditions (Fig. 3 *C*). Moreover, lattice compression by 1–3% (w/v) dextran had no significant effect on probe orientation in the presence of 1 μmol L^−1^ OM at either pCa 9 or pCa 4.3 (data not shown). Neither activation of isometric force by OM at pCa 9 nor inhibition of force at pCa 4.3 are mediated by changes in thin filament structure as reported by the TnC E‐helix probe.

At 5 μmol L^−1^ BS did reduce the level of thin filament activation at pCa 4.3 reported by the cTnC E‐helix probe by ∼30% (Fig. 5 *D* and Table 1), in agreement with previous results during almost complete force inhibition at 25 μmol L^−1^ BS (Sun *et al*. 2009). However, increasing [BS] to 25 μmol L^−1^ in the present experiments had no further effect on cTnC E‐helix probe orientation at pCa 4.3 (squares in Fig. 5 *D*), despite the further inhibition of isometric force from ∼25% to ∼1%. Thus, as in the case of OM, the effects of BS on isometric force are not simply related to thin filament structure as monitored by the TnC E‐helix probe.

The calcium sensitivity (pCa_50_) of the change in thin filament activation reported by the cTnC E‐helix probe was increased by 1 μmol L^−1^ OM and decreased by 5 μmol L^−1^ BS (Figs 5 *D* and 6 *A*), consistent with the hypothesis that the activation state of the thin filament is sensitive to that of the thick filament at physiological [Ca^2+^] (Kampourakis *et al*. 2016). OM (1 μmol L^−1^) reduced the steepness (*n*_H_) of the calcium dependence of TnC E‐helix probe orientation to 2.5 (Fig. 6 *B*), similar to the effect on force and thick filament structure reported by the cRLC probe (Figs 5 *B* and 6 *B*), but BS had a smaller effect on *n*_H_ for TnC E‐helix probe orientation, which was still 4.2 in the presence of 5 μmol L^−1^ BS (Fig. 6 *D*, Table 1). The effect of 1 μmol L^−1^ OM on pCa_50_ for TnC probe orientation was significantly smaller than that for force, in contrast with its effect on pCa_50_ for the cRLC probe in the thick filament, which was larger than that for force (Fig. 6 *A*).

## Discussion

### OM and BS stabilize ON and OFF states of the *thick* filament respectively in the absence of Ca^2+^

In this study we have for the first time established detailed structure–function relationships for OM and BS that integrate functional, biochemical and structural measurements in the native environment of the intact muscle lattice. Our results establish a novel mechanistic basis for the modulation of myosin function via thick filament‐based regulation and coupling between the regulatory states of the thick and thin filaments, and suggest how those mechanisms contribute to the normal physiological regulation of contractility in the heart.

We have interpreted the antagonistic effects of OM and BS on the orientation of the myosin cRLC in terms of structural OFF and ON states of the thick filament. Those states were monitored using a probe that has its fluorescence dipole parallel to the cRLC E‐helix (Kampourakis *et al*. 2014, 2015, 2016) (Fig. 1). This probe is more parallel to the thick filament axis in the myosin OFF state or interacting heads motif (IHM) observed in EM reconstructions of isolated thick filaments (Al‐Khayat *et al*. 2013) and this orientation is measured as a higher value of the order parameter <*P*_2_>. The results from this cRLC E‐helix probe showed that, at very low [Ca^2+^] (pCa 9), OM induces a more ON state of the thick filament, whereas BS induces a more OFF state (Fig. 3). This conclusion is consistent with the contrasting effects of OM and BS on the ATPase of isolated myosin head domains (Kovacs *et al*. 2004; Malik *et al*. 2011; Liu *et al*. 2015; Swenson *et al*. 2017); OM accelerates the rate of release of inorganic phosphate (P_i_) from the myosin.ADP.P_i_ intermediate, whereas BS inhibits it. Thus OM increases the fraction of myosins in the ADP state that can bind strongly to actin and generate force, whereas BS increases the fraction in the weak‐binding or low‐force ADP.P_i_ state. These two states are associated with different conformations of the myosin head domain, the switch‐2 open and closed states, respectively. Moreover, the ADP.P_i_ or switch‐2 closed conformation is required for the OFF structure of the thick filament (Xu *et al*. 1999; Zoghbi *et al*. 2004; Zhao *et al*. 2008). Furthermore, the quantitative effect of both compounds (OM and BS) is most likely modulated by post‐translational modifications of proteins associated with regulatory structural transitions in the thick filament, e.g. phosphorylation of cRLC and cMyBP‐C.

The biochemical correlate of the myosin OFF state has been described for both skeletal and cardiac muscle, and termed the super‐relaxed state (SRX), characterized by a population of myosin heads with ultra‐low ATPase activity (Stewart *et al*. 2010; Hooijman *et al*. 2011). Structural and functional studies in isolated skeletal muscle fibres (Wilson *et al*. 2014) and human myocardium (Tang *et al*. 2016) suggested that BS and its derivatives stabilize the SRX.

**Figure 7:**
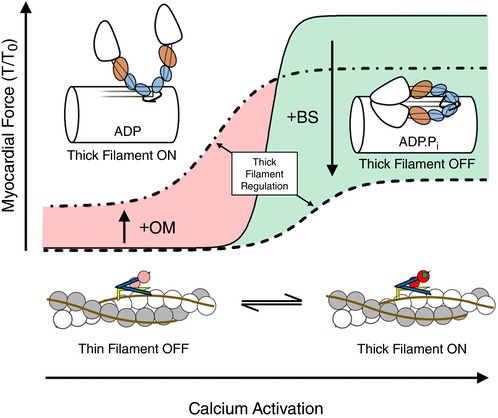
Proposed model for the effects of OM and BS on myofilament Ca^2+^ activation Under control conditions in the absence of any drug, normalized myocardial force shows a steep dependence on [Ca^2+^] (continuous line). OM induces a perpendicular myosin head orientation, activates the thick filament in the absence of Ca^2+^ and stabilizes the myosin.ADP state (left). Thick filament activation by OM increases myocardial calcium sensitivity of force, but decreases cooperativity of activation (dashed‐dotted line). In contrast, BS stabilizes the thick filament OFF state characterized by a parallel myosin head orientation in the ADP.P_i_ state (right), and decreases calcium sensitivity and cooperativity of force (dashed line). [Color figure can be viewed at wileyonlinelibrary.com]

Consistent with these structural and biochemical correlations, OM and BS have opposite effects on isometric force at pCa 9; OM activates force and BS inhibits it (Fig. 2). The ability of OM to activate force in the absence of calcium, when the thin filament would normally be switched OFF, implies that myosin heads with bound OM can bind to thin filaments and displace tropomyosin to its ON position in the absence of calcium binding to troponin (Fig. 7), consistent with the decreased rate of relaxation observed in isolated cardiomyocytes in the presence of OM (Nagy *et al*. 2015).

However, the orientation of the cTnC‐E helix probe did not change when the thick filament and isometric force were activated by OM in these conditions (Fig. 5), in marked contrast to the large change in cTnC E‐helix probe orientation associated with calcium activation and the even larger change associated with activation by rigor myosin heads (Sun *et al*. 2009). Although all these thin filament activation pathways presumably involve a change in the azimuthal position of tropomyosin, activation by OM appears to work through a distinct inter‐filament signalling pathway which is not directly coupled to a change in the cTnC E‐helix orientation. Activation by OM at pCa 9 is also associated with a modest decrease in the myofibrillar ATPase activity, in contrast with the ∼15‐fold increase associated with calcium activation (Fig. 4), and this is presumably associated with direct inhibition of ATPase in myosin heads with bound OM.

### Effects of OM and BS in the presence of calcium reveal regulatory coupling between thick and thin filaments

The competing activating and inhibitory effects of OM are modified in the presence of calcium. At full calcium activation (pCa 4.3) OM strongly inhibits isometric force (Figs 2 *A* and 3 *A*) and cross‐bridge kinetics (Mamidi *et al*. 2015), and modestly reduces myofibrillar ATPase (Fig. 4), despite inducing a more ON state of the thick filament (Fig. 3 *C*). These inhibitory and activating effects have the same EC_50_, suggesting that both are the consequence of OM binding to a single site on the myosin head to stabilize a myosin.ADP state (Rohde *et al*. 2017; Swenson *et al*. 2017). Similar to results in the absence of calcium discussed above, OM binding to myosin does not alter the regulatory state of the *thin* filament as reported by the TnC E‐helix probe at pCa 4.3 (Fig. 5). As in that case, we conclude that the competing activating and inhibitory effects of OM at pCa 4.3 are mediated by changes in thick filament structure and actin‐activated myosin ATPase, respectively. The activating effect dominates contractility at low [OM] and [Ca^2+^], probably as a result of the co‐operativity of thick filament activation, with a Hill co‐efficient for [OM] dependence of 1.75, whereas the inhibitory effect dominates at higher [OM] and [Ca^2+^], when both thick and thin filaments are largely ON. These competing effects may be responsible for the bell‐shaped dependence of isometric force on [OM] at low and intermediate [Ca^2+^] (Figs 2 *A* and 3 *A*).

BS, in contrast, stabilizes the OFF structure of the thick filament and inhibits force production (Fig. 3) and myofibrillar ATPase in all conditions studied. The effects of BS on active force and thick filament structure have the same EC_50_, consistent with both being associated with stabilization of the ADP.P_i_ state of the myosin head presumably in the pre‐power‐stroke conformation (Alamo *et al*. 2017), reducing ATP turnover and promoting the OFF structure of the thick filament. BS had no effect on the level of activation of the *thin* filament as reported by the cTnC E‐helix probe at pCa 9, but reduced its activation level at pCa 4.3 (Fig. 5), indicating positive coupling between the activation states of the thick and thin filaments in the presence of calcium.

Although OM does not affect the regulatory state of the thin filament reported by the cTnC E‐helix probe at either pCa 9 or 4.3, at intermediate [Ca^2+^] in the physiological range it switches ON the thin filament as well as the thick filament, and BS switches OFF both filaments (Figs 5 and 6). Both small molecules can perturb the normal coupling between the regulatory states of the thick and thin filaments, as shown by the altered Ca^2+^ dependence of structural changes in the myofilaments and force development. In the absence of either OM or BS, activation of the thick and thin filaments and isometric force have similar but not identical [Ca^2+^] dependence; calcium sensitivity (pCa_50_) is close to 5.5, and Hill coefficients (*n*_H_) are greater than 5.5 (Fig. 6; Table 1). However, *n*_H_ for thick filament structure is significantly higher than that for thin filament structure or force, as reported previously for a different probe on the N‐lobe of the cRLC (Kampourakis *et al*. 2016); calcium activation of the thick filament is more co‐operative than that of the thin filament. Co‐operativity of the thin filament is likely to be associated with end‐to‐end joining between tropomyosin regulatory units (Gordon *et al*. 2000); that of the thick filament to the multiple inter‐ and intra‐molecular interactions that stabilize the thick filament OFF state (Al‐Khayat *et al*. 2013).

The effects of BS and OM reported above have implications for the roles of thin and thick filament co‐operativity during normal contractile activation. When the thick filament is locked in its OFF state by 5 μmol L^−1^ BS, so that its structure becomes independent of [Ca^2+^] (Fig. 5 *B*), calcium activation becomes less co‐operative – *n*_H_ for force is reduced from more than 6 to less than 3, and calcium sensitivity pCa_50_ is reduced by about 0.25 pCa units (Table 1). Pushing the thick filament towards the ON state with 1 μmol L^−1^ OM also reduces *n*_H_ to less than 3, but *increases* calcium sensitivity of force by about 0.3 pCa_50_ units (Figs 5 and 6).

These effects suggest that the intrinsic co‐operativity of the thin filament activation is low, and that its calcium sensitivity depends on the regulatory state of the *thick* filament (Fig. 7). When the thick filament is OFF, pCa_50_ is about 5.3 (as in the presence of BS; Fig. 7, dashed line); when the thick filament is ON, pCa_50_ is about 5.5 or higher (as in the presence of OM; Fig. 7, dashed‐dotted line). In physiological conditions, the thick filament is OFF at low [Ca^2+^], and no active force is produced at pCa > 5.9. At slightly higher [Ca^2+^], around pCa 5.7, the thin filament starts to be activated and this starts to activate the thick filament, which in turn increases the calcium sensitivity of the thin filament (Fig. 7, red shaded area). The net result of this inter‐filament coupling is to produce a very steep dependence of the activation state of both filaments and isometric force on [Ca^2+^] (continuous line).

Taken together, our results suggest that the force–calcium relation of cardiac muscle is controlled by three regulatory pathways working in parallel: calcium activation of the thin filament, the regulatory state of the thick filament and intermediates in the acto‐myosin ATPase pathway.

This scheme also provides a qualitative explanation for the effects of other interventions that affect the regulatory state of the thick filament, like RLC phosphorylation and increased sarcomere length (Kampourakis *et al*. 2016), on the calcium sensitivity of the thin filament and isometric force. Moreover, hypertrophic and dilated cardiomyopathy mutations have been shown to alter the calcium dependence of myofilament activation in a similar manner to OM and BS (Spudich, 2014), implicating disturbed inter‐filament coupling in the aetiology of cardiomyopathy‐associated heart failure. An uncoupling between the regulatory structural changes in the thick filament and force generation similar to that produced by OM has been recently suggested as a mechanism underlying the hyper‐contractility associated with hypertrophic cardiomyopathy mutations in the myosin head domain (Alamo *et al*. 2017; Nag *et al*. 2017; Trivedi *et al*. 2017).

The molecular mechanisms responsible for coupling between the regulatory states of the thin and thick filaments remain to be elucidated, but candidate mechanisms would include binding of myosin heads to thin filaments (Gordon *et al*. 2000) and binding of the N‐terminus of cMyBP‐C to the thin filaments (Pfuhl & Gautel, 2012; Kampourakis *et al*. 2014). Binding of myosin heads to thin filaments stabilizes their ON state by preventing tropomyosin moving back to its OFF position, so an ON thick filament can promote an ON thin filament. Similarly, binding of the N‐terminus of cMyBP‐C to the thin filaments also stabilizes their ON state, although it interacts with myosin head domains with similar affinity, and also has an inhibitory effect on the thick filament structure (Pfuhl & Gautel, 2012; Kampourakis *et al*. 2014; Mun *et al*. 2014). The major rearrangement of the myosin heads associated with activation of the thick filament might disrupt an interaction between cMyBP‐C and myosin heads, promoting a competing activating interaction with the thin filament (Kampourakis *et al*. 2014). Reciprocal (thin to thick) effects are also possible and additional mechanisms may contribute to inter‐filament signalling. Thick filament mechano‐sensing (Linari *et al*. 2015), for example, postulates that the regulatory state of the thick filament is controlled by mechanical stress in the filament, which is itself a consequence of force generation and therefore of thin filament activation. This mechanism would also generate positive co‐operativity, but requires a population of myosin heads outside thick filament control to initiate the positive feedback loop.

Further consideration of the relative significance of these and other potential mechanisms of interfilament signalling is likely to require a more detailed understanding of the structural basis of the regulatory states of the thin and thick filaments, at a level beyond the simple concept of OFF and ON states. For the thin filaments, the distinct responses of probes on the cTnC C and E‐helices to BS (Sun *et al*. 2009; Kampourakis *et al*. 2014) provide further support for a concept of thin filament regulation in the intact sarcomere as a signalling pathway in which different structural components may be activated to different extents by the different inputs to the pathway, including calcium, post‐translational modifications, binding of myosin heads and the actions of cMyBP‐C. Thick filament regulation is also likely to constitute an analogous multi‐component pathway with multiple inputs for which simple OFF and ON descriptions are an oversimplification. Moreover, different regions of the thick filament, for example the C‐zone containing cMyBP‐C, are likely to have distinct regulatory states. Finally, it will be important to extend the present consideration of steady‐state regulatory states to the dynamic switching that underlies the cardiac cycle.

### Therapeutic mechanism of OM and implications for drug design

Inherited cardiomyopathies including hypertrophic, dilated and restricted cardiomyopathy are commonly linked to mutations in the protein components of the thick and thin filaments, and filament dysfunction is a major cause of heart failure (Morita *et al*. 2010; McNally *et al*. 2013). Available treatments for all these types of heart disease are clearly inadequate, and much effort continues to be directed towards establishing more effective therapies with reduced off‐target effects. Drugs that enhance contractility by boosting the calcium transient suffer from the consequences of the multiple intracellular targets for calcium, and altered calcium handling can induce arrhythmias. This limitation led to an increased emphasis on targeting the thick and thin filaments themselves, initially focused on troponin in the thin filament, which was perceived to be the primary site of regulation of contraction (Malik *et al*. 2011; Radke *et al*. 2014; Spudich, 2014; Hwang & Sykes, 2015; Green *et al*. 2016). More recently attention has been directed towards myosin itself (Malik *et al*. 2011; Radke *et al*. 2014; Spudich, 2014; Green *et al*. 2016), and the search for myosin‐targeted therapeutics has been directed by biochemical and structural models of the interaction between the myosin head domain, actin and ATP, which have been used to screen for and characterize the function of small molecules that bind specifically to cardiac myosin, and activate (Malik *et al*. 2011), inhibit (Green *et al*. 2016), or restore (Radke *et al*. 2014) its function. Omecamtiv mecarbil (OM), the activator used in the present study, has completed phase II clinical trials for the treatment of systolic heart failure (Teerlink *et al*. 2016).

The results presented above showed that, at clinically relevant concentrations, OM activates cardiac muscle cells by disrupting the OFF state of the thick filament, which in turn increases the calcium sensitivity of the thin filament, producing a large increase in contractility at physiological calcium concentrations. OM also inhibits the ATPase activity of myosin and this inhibitory effect becomes dominant at higher [OM] and [Ca^2+^]. Both the activating and inhibitory effects of OM are mediated by stabilization of a myosin.ADP state, and the therapeutic window of [OM] and [Ca^2+^] is created by the intrinsic co‐operativity of the thick and thin filament regulatory mechanisms.

Our results also suggests a novel approach to the development of myosin‐targeted small molecules that modulate the contractility of cardiac muscle, either positively, to enhance the performance of the failing heart, or negatively, as might be useful in the treatment of diastolic failure associated with hypertrophic cardiomyopathy. To supplement the approach of looking for small molecules like OM and BS that perturb the actin‐myosin ATPase cycle as determined with soluble myosin fragments that do not form filaments, it might be possible to design or screen for molecules that perturb the regulatory state of the thick filament without large effects on the actin‐myosin ATPase (Nogara *et al*. 2016a). In principle, and in contrast with OM, such molecules could be simple activators without competing inhibitory effects, which might facilitate the selection of a therapeutic dose.

## Additional information

### Competing interests

The authors declare no competing financial interests.

### Author contributions

Conception and design: T.K., Y.B.S., M.I. Acquisition, analysis and interpretation of data: T.K., X.Z., M.I. Manuscript preparation: T.K., M.I. All authors have approved the final version of the manuscript. All authors agree to be accountable for all aspects of the work in ensuring that questions related to the accuracy or integrity of any part of the work are appropriately investigated and resolved. All persons designated as authors qualify for authorship, and all those who qualify for authorship are listed.

### Funding

This work was supported by the British Heart Foundation (PG/12/52/29713, FS/16/3/31887, FS/09/001/26329).
